# Intubated COVID-19 predictive (ICOP) score for early mortality after intubation in patients with COVID-19

**DOI:** 10.1038/s41598-021-00591-1

**Published:** 2021-10-26

**Authors:** Mitsuaki Nishikimi, Rehana Rasul, Cristina P. Sison, Daniel Jafari, Muhammad Shoaib, Koichiro Shinozaki, Timmy Li, Kei Hayashida, Daniel M. Rolston, Jamie S. Hirsch, Lance B. Becker, The Northwell Health COVID-19 Research Consortium, Matthew A. Barish, Douglas P. Barnaby, Santiago J. Miyara, Edith Burns, Stuart L. Cohen, Jennifer Cookingham, Andrew J. Dominello, Jennifer C. Johnson, Zachary M. Kozel, Brian Lima, Ariana K. McGinn, Ernesto P. Molmenti, Rachel Monane, Marc d. Paradis

**Affiliations:** 1 Laboratory of Critical Care Physiology, Feinstein Institutes for Medical Research, Northwell Health, 350 Community Dr, Manhasset, NY 11030 USA; 2 Biostatistics Unit, Feinstein Institutes for Medical Research, Northwell Health, Manhasset, NY USA; 3 Donald and Barbara Zucker School of Medicine at Hofstra/Northwell, Hempstead, NY USA; 4 Department of Surgery, North Shore University Hospital, Northwell Health, Manhasset, NY USA; 5 Department of Emergency Medicine, North Shore University Hospital, Northwell Health, Manhasset, NY USA; 6 Institute of Health Innovations and Outcomes Research, Feinstein Institutes for Medical Research, Northwell Health, Manhasset, NY USA; 7 Department of Information Services, Northwell Health, New Hyde Park, NY USA; 8 North Shore University Hospital/Northwell Health, Manhasset, NY USA; 9 Institute of Health Innovations and Outcomes Research, The Feinstein Institutes for Medical Research, Northwell Health, Manhasset, NY USA; 10 Laboratory of Critical Care Physiology, The Feinstein Institutes for Medical Research, Northwell Health, Manhasset, NY USA; 11 Elmezzi Graduate School of Molecular Medicine, Manhasset, NY USA; 12 Department of Data Strategy & Ventures, Northwell Health, Manhasset, NY USA

**Keywords:** Risk factors, Viral infection, Respiratory distress syndrome

## Abstract

Patients with coronavirus disease 2019 (COVID-19) can have increased risk of mortality shortly after intubation. The aim of this study is to develop a model using predictors of early mortality after intubation from COVID-19. A retrospective study of 1945 intubated patients with COVID-19 admitted to 12 Northwell hospitals in the greater New York City area was performed. Logistic regression model using backward selection was applied. This study evaluated predictors of 14-day mortality after intubation for COVID-19 patients. The predictors of mortality within 14 days after intubation included older age, history of chronic kidney disease, lower mean arterial pressure or increased dose of required vasopressors, higher urea nitrogen level, higher ferritin, higher oxygen index, and abnormal pH levels. We developed and externally validated an intubated COVID-19 predictive score (ICOP). The area under the receiver operating characteristic curve was 0.75 (95% CI 0.73–0.78) in the derivation cohort and 0.71 (95% CI 0.67–0.75) in the validation cohort; both were significantly greater than corresponding values for sequential organ failure assessment (SOFA) or CURB-65 scores. The externally validated predictive score may help clinicians estimate early mortality risk after intubation and provide guidance for deciding the most effective patient therapies.

## Introduction

Coronavirus disease 2019 (COVID-19) was designated as a global pandemic in March 2020 by the World Health Organization. By September 2020, there were over 32 million people globally with COVID-19 and approximately 985 000 deaths. The United States has surpassed all other countries in total cases (almost 7 million) and deaths (over 202 000)^1,2^. Approximately 16% of the patients infected with COVID-19 showed severe acute respiratory failure^1^, and 4–12% needed invasive respiratory support^3,4^.

The in-hospital mortality rate of intubated COVID-19 patients worldwide ranges from approximately 8% to 67%^5,6^, but in the US, it is between 23 and 67%^5^. There is substantial variability in the disease process, such that some patients rapidly deteriorate and die of severe respiratory failure or multiple organ failure within 1 to 2 weeks after intubation, while others recover, despite requiring mechanical ventilation. Stratifying the risk of further deterioration in these patients serves to help guide medical providers and family members in joint decision-making about the future treatment options of the patients, as well as to stratify patients in future epidemiologic/therapeutic studies for COVID-19^7^. To date, many independent factors used to predict mortality of admitted COVID-19 patients have been reported^8–11^, but none are designed exclusively for intubated patients—the most severely ill population with the highest risk of death. Also, a recent study showed sequential organ failure assessment (SOFA) score, which has been typically used for estimating severity of intubated patients, did not show acceptable predictive accuracy (area under the curve [AUC] = 0.59), which means a better prediction tool for this population is needed^12^.

Identifying patients with a high risk of early phase mortality after intubation is clinically important because these patients may require more aggressive therapeutic strategies, such as rescue therapy with extracorporeal membrane oxygenation (ECMO)^13^. In some cases, patients need to be transferred to tertiary hospitals as soon as possible where these treatments are available. Therefore, the aim of this study was to develop a predictive score that could identify COVID-19 patients with high risk of early mortality after intubation.

## Material and methods

This retrospective cohort study was conducted at hospitals in Northwell Health, the largest academic health system in New York, serving approximately 11 million people annually. The Northwell Health Institutional Review Board approved this study as minimal-risk research using data collected for routine clinical practice and waived the requirement for informed consent. All the methods were performed in accordance with all the relevant guidelines and regulations.

The study included all patients (18 years or older) who required hospitalization and intubation and with confirmed severe acute respiratory syndrome coronavirus 2 (SARS-CoV-2) infection between March 1, 2020 and April 27, 2020. COVID-19 was confirmed by a positive result on polymerase chain reaction (PCR) testing of a nasopharyngeal sample. Patients were admitted to one of 12 Northwell Health hospitals, and clinical outcomes were monitored until July 20, 2020, the final follow-up date.

### Data collection

Data were collected from our health system’s electronic medical record (EHR; Sunrise Clinical Manager; Allscripts, Chicago, IL, U.S.) reporting database. Manual chart reviews were conducted by research staff and medical students.

Data collected included patient demographic information (age, sex, race, insurance type, language), comorbidities, laboratory test results and vital signs at the time of intubation, diagnoses during the hospital course, treatments, and outcomes (7-, 14-, and 28-day mortality after intubation). Race was self-reported in pre-specified fixed categories in the electronic medical record. The primary outcome was in-hospital mortality within 14 days after intubation. Mortality within 28 days after intubation was the secondary outcome. For analyses on 28-day mortality after intubation, patients were excluded if they were transferred out of the Northwell Health system before 28 days.

Patients were excluded if they were less than 18 years old at time of admission, died within 24 h after intubation, transferred to a hospital outside of Northwell Health system within 14 days after intubation, transferred into the Northwell Health system from outside hospitals after intubation, or were placed on ECMO.

We separated the data for the derivation and validation cohorts based on region to perform narrow external validation^14^, with New York City and Suffolk County cases in the derivation sample and Nassau County cases in the validation sample. All methods for separating the cohorts, variable selection, imputation for missing values, and statistical modeling to determine the predictive score were decided before the development of the analytic datasets. Additional information for the statistical analyses performed is provided in the eMethods in the Supplementary material.

### Model construction

Candidate predictors, chosen from clinical variables which can be easily obtained in the early phase after intubation, and the proportion of missing data for each variable were summarized in eTable 1 in the Supplementary material. Multiple imputation was performed, assuming data were missing at random, for candidate predictors with < 25% missing values, using predictive mean matching for continuous variables and discriminant analysis for categorical variables. Logistic regression models starting with all 36 candidate predictors were fitted to determine the predictive score using each of the 33 imputed datasets from the derivation cohort. Backwards selection using *P* < 0.01 and *P* < 0.05 was applied for variable selection. The selection criterion yielding the fewest candidate predictors with minimal loss of discrimination was then selected. Logistic regression was again performed for each imputed dataset using variables appearing in at least half of the models. Rubin’s rules were used to calculate pooled estimates and standard errors^15^.

### Development of the simplified score

A logistic regression was performed for each imputed dataset using all selected variables from the model construction and estimates were pooled using Rubin’s rules. If a selected variable was continuous, it was categorized before inclusion in the model. For each variable, points equivalent to the odds ratio corresponding to the group to which the patient belonged, were rounded to the nearest integer and assigned to each patient. A patient in a reference group was assigned 0 points. The simplified score, sICOP, was calculated as the sum of the points across all variables for the patient.

### Model performance

Model performance of the predictive score as well as the simplified score was assessed using discrimination and calibration. Discrimination was quantified as the area under the receiver operating characteristic curve (*c*-statistic). To simplify reporting, predicted probabilities were averaged across the imputed datasets to calculate the c-statistic. Calibration was assessed by plotting the agreement between observed outcomes and predicted probabilities.

### Internal and external validation

Both the predictive score and simplified score were internally validated using the optimism-corrected *c*-statistic. The derivation cohort was bootstrapped 100 times with replacement and each bootstrapped dataset was imputed 33 times, resulting in 3300 imputed datasets. Model selection was performed in each of the 3300 imputed datasets using backward selection. Optimism was calculated as the average of the differences between the *c*-statistic from each model and the *c*-statistic from the bootstrapped model when applied to the original dataset. The validation cohort was imputed 33 times using similar procedures described for the derivation cohort. The *c*-statistics of our new predictive scores in both the derivation and validation cohorts were compared to the corresponding values for the SOFA score and the CURB-65 score using Delong’s test^16^. In the comparison with SOFA score or CURB-65 score, only patients in whom all variables needed for the calculation of the corresponding scores were available, were analyzed. We used the guidelines for reporting and analysis from the Transparent Reporting of multivariable prediction model for Individual Prognosis or Diagnosis (TRIPOD) statement^14^. All analyses were performed using SAS 9.4 (SAS Institute Inc., Cary, NC).

### Ethics approval and consent to participate

Ethics approval was obtained by the Institutional Review Board of Northwell Health.

### Consent to publish

The Northwell Health institutional review board approved this study as minimal-risk research using data collected for routine clinical practice and waived the required for informed consent.

## Results

**Figure 1:**
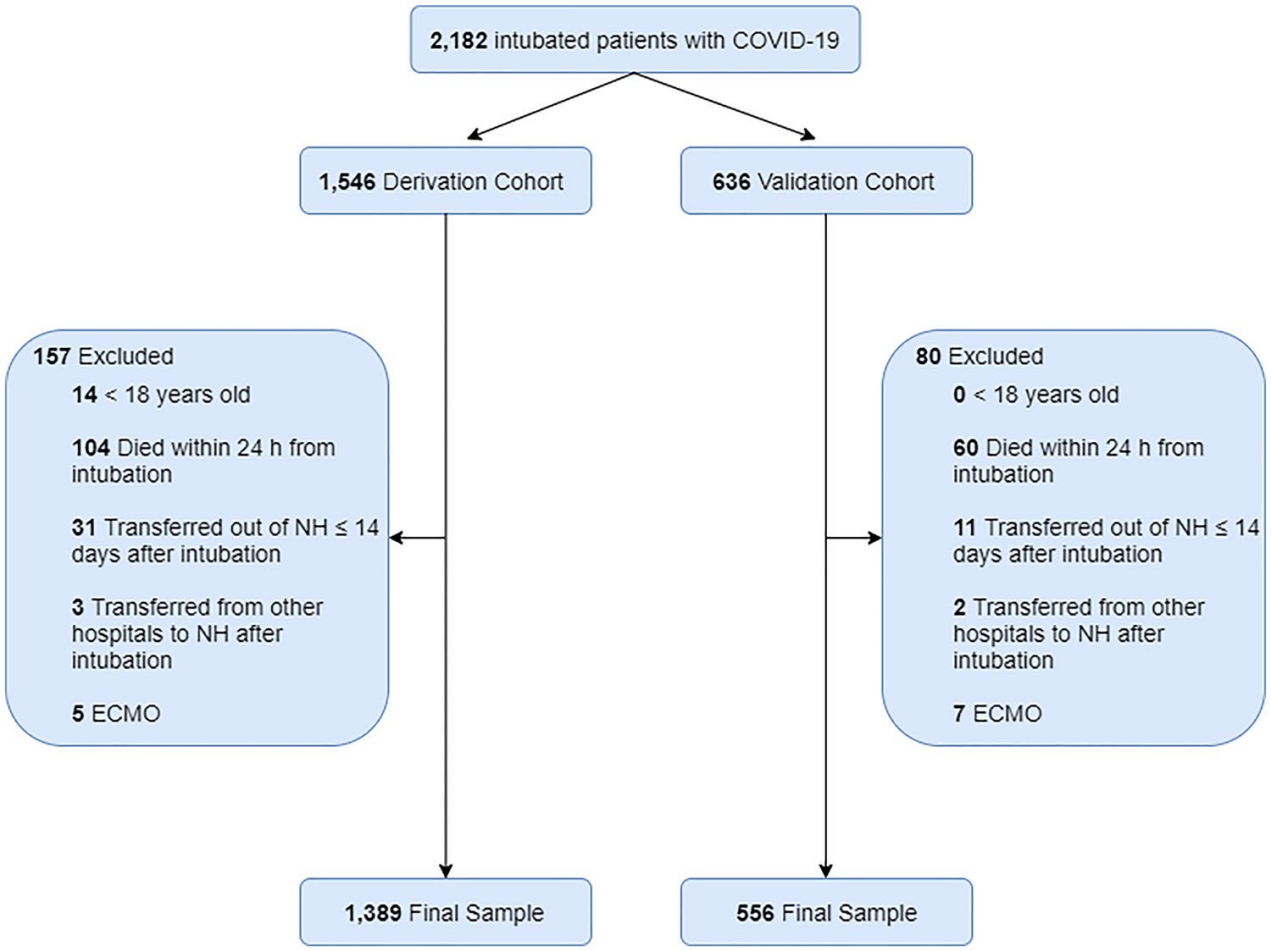
Flow diagram of patients. *NH*, Northwell Health; *ECMO*, extracorporeal membrane oxygenation.

A total of 2182 adult intubated patients with COVID-19 admitted to all hospitals in Northwell Health system were included (n = 1546 for the derivation cohort and n = 636 for the validation cohort). Among them, 157 and 80 were excluded, respectively, because they were under 18 years old (n = 14 and n = 0), died within 24 h after intubation (n = 104 and n = 60), transferred outside of the Northwell Health system within 14 days after intubation (n = 31 and n = 11), transferred from hospitals outside of the Northwell Health system after intubation (n = 3 and n = 2), or placed on ECMO (n = 5 and n = 7). The data of the remaining 1389 and 556 patients were analyzed in this study, respectively (Fig. 1). Characteristics of the derivation and validation cohorts, such as age, sex, race, comorbidities and treatments, are indicated in Table 1.

**Table 1:** Characteristics of adults with coronavirus disease 2019 presenting to 12 hospitals in the greater New York City area.

	Derivation cohort	Validation cohort
**Demographic information**		
Total no	1389	556
Age, median (IQR), y	65 (56–73)	67 (58–75)
Sex, male, n (%)	964 (69.4)	366 (65.8)
BMI, median (IQR)^a^	29 (26–34)	29 (25–34)
Race^b^, n (%)		
Asian	129 (9.3)	66 (11.9)
Black	223 (16.1)	119 (21.4)
White	499 (35.9)	211 (38.0)
Other/multiracial	467 (33.6)	142 (25.5)
Unknown	71 (5.1)	18 (3.2)
Insurance, n (%)		
Commercial	427 (30.7)	161 (29.0)
Medicaid	303 (21.8)	113 (20.3)
Medicare	610 (43.9)	272 (48.9)
Other^c^/self pay	49 (3.5)	10 (1.8)
Language, n (%)		
English	1032 (74.3)	445 (80.0)
Other	357 (25.7)	111 (20.0)
Time from admission until intubation, median (IQR), days	1.94 (0.3–5.1)	2.17 (0.2–5.5)
Intubation at pre-hospital or ER	188 (13.5)	67 (12.1)
**Comorbidities, n (%)**		
Hypertension	849 (61.1)	346 (62.2)
Diabetes	564 (40.6)	244 (43.9)
Heart disease	385 (27.7)	173 (31.1)
Lung disease	237 (17.1)	99 (17.8)
Cancer	131 (9.4)	65 (11.7)
Dementia	54 (3.9)	19 (3.4)
CKD	120 (8.6)	46 (8.3)
Chronic liver diseases	27 (1.9)	14 (2.5)
**Vital signs, median (IQR)**^**a**^		
Heart rate, beats/min^a^	106 (89–122)	110 (94–126)
Mean arterial pressure, mmHg	77 (67–90)	75 (65–88)
Urinary OUTPUT, ml/kg/h	3.2 (1.5–5.7)	3.2 (1.4–5.5)
**Laboratory values, median (IQR)**^**a**^		
Albumin, g/dL	2.9 (2.5–3.3)	2.7 (2.3–3.1)
ALP, U/L	86.5 (64–123)	93 (64–138)
Total bil, mg/dL	0.6 (0.4–0.9)	0.6 (0.4–0.8)
Total protein, g/dL	6.8 (6.2–7.4)	7 (6.4–7.6)
BUN, mg/dL	26 (16–43)	27 (17–44)
Creatinine, mg/dL	1.2 (0.8–1.8)	1.1 (0.8–1.9)
CRP, mg/L	15.54 (8.16–25.38)	13.9 (6.78–23.52)
d-dimer, × 10^3^ μg/ml	1.2 (0.6–4.2)	1.2 (0.6–3.9)
Ferritin, × 10^3^ ng/ml	1.1 (0.7–2.1)	1.1 (0.7–2.3)
Hematocrit, %	39 (35–43)	39 (35–44)
NLR^a^	12.4 (7.6–21.6)	11.7 (7.3–21.5)
Plat count, 10^5/^/μL	2.4 (1.8–3.3)	2.4 (1.8–3.4)
Potassium, mmol/L	4.3 (3.9–4.8)	4.2 (3.8–4.8)
Procalcitonin, ng/mL	0.6 (0.2–1.5)	0.5 (0.2–1.5)
RCDW, %	13.9 (13.2–14.9)	14.1 (13.2–15.2)
Sodium, mmol/L	138 (135–142)	138 (134–142)
WBC Count, K/μL	11.9 (8.6–16.7)	12.3 (8.5–18.2)
Lactate, mmol/L	1.7 (1.2–2.6)	2 (1.4–3.2)
**Blood gases, median (IQR)**^**a**^		
AaDO_2_	487 (375–554)	507 (402–554)
PF ratio	131 (87–203)	117 (86–176)
Oxygen index	13 (9–21)	15 (9–21)
pH	7.3 (7.2–7.4)	7.3 (7.2–7.3)
**Treatment, n (%)**		
Steroid therapy	1127 (81.2)	462 (83.1)
Anticoagulant therapy	1376 (99.1)	553 (99.5)
CHDF treatment	319 (23.0)	165 (29.7)
**Outcome, n (%)**		
7-day mortality	386 (27.8)	175 (31.5)
14-day mortality	608 (43.8)	267 (48.0)
28-day mortality^d^	832 (60.7)	369 (66.4)

IQR, interquartile range; BMI, body mass index; ER, emergency department; CKD, chronic kidney disease; ALP, alkaline phosphatase; Bil, bilirubin; BUN, blood urea nitrogen; CRP, C-reactive protein; NLR, neutrophil to lymphocyte ratio; Plat, platelet; RCDW, red blood cell distribution width; WBC, white blood cell; CHDF, continuous hemodiafiltration.

^a^Missing data is summarized in eTable 1 in the Supplement.

^b^Race was collected by self-report in prespecified fixed categories.

^c^Other insurance includes military, union, and workers’ compensation.

^d^We performed analysis by using data from 1389 patients in the derivation cohort and 556 patients in the validation cohort.

The 14-day mortality rate was 43.8% (608/1389) in the derivation and 48.0% (267/556) in the validation cohort. A total of 36 factors were considered as candidate predictors for developing the predictive score (eTable 2 in the Supplementary material). After the model selection process, 7 variables were selected (age, past medical history of chronic kidney disease [CKD], the values of mean arterial pressure [MAP]/needed dose of vasopressors, oxygen index [OI], the laboratory values of blood urea nitrogen [BUN], ferritin, and pH) (eFigure 1 in the Supplementary material). Increased age, and values of OI, BUN, and ferritin were associated with increased odds of early mortality after intubation, as well as the past medical history of CKD. Abnormal levels of pH were also associated with increased odds of early mortality compared to normal levels. Patients who required dopamine > 15 µg/kg/min or epinephrine > 0.1 µg/kg/min, or those who required epinephrine > 0.2 µg/kg/min or norepinephrine > 0.2 µg/kg/min were associated with increased odds of early mortality compared to those whose MAP was ≥ 70 mmHg (Table 2).

**Table 2:** Pooled results of multivariable logistic regressions over 33 imputed datasets.

Predictor	Coefficient	OR (95% CI)	*P* value
Intercept	− 4.226521	–	< 0.001
Age, y	0.036078	1.04 (1.03–1.05)	< 0.001
Past medical history of CKD	0.664208	1.94 (1.24–3.05)	0.004
BUN, mg/dL	0.014718	1.01 (1.01–1.02)	< 0.001
Ferritin, × 10^3^ ng/ml	0.071151	1.07 (1.02–1.12)	0.009
OI	0.026700	1.31 (1.14–1.50)	< 0.001
**pH (ref = > 7.30 to ≤ 7.40)**			
≤ 7.10	0.991712	2.70 (1.63–4.45)	< 0.001
> 7.10 to ≤ 7.20	0.594503	1.81 (1.22–2.69)	0.003
> 7.20 to ≤ 7.30	0.423305	1.53 (1.12–2.08)	0.007
> 7.40	0.128661	1.14 (0.80–1.62)	0.48
**MAP/dose of needed vasopressor (ref = MAP ≥ 70)**			
MAP < 70, no vasopressor	0.068626	1.07 (0.75–1.52)	0.70
DOA ≤ 15 r/ EPI or NAD ≤ 0.1 r	0.303636	1.35 (0.96–1.92)	0.09
DOA > 15 r/EPI or NAD ≤ 0.2 r	0.645441	1.91 (1.20–3.02)	0.006
EPI or NAD > 0.2 r	1.106242	3.02 (1.95–4.68)	< 0.001

y, years; CKD, chronic kidney disease; BUN, blood urea nitrogen; OI, oxygen index; Ref, reference level; OR, odds ratio; CI, confidence interval; MAP, mean arterial pressure; r, mg/kg/min; DOA, dopamine; EPI, epinephrine; NAD, norepinephrine.

The predictive score, hereby designated Intubated COVID-19 Predictive score (ICOP score), is the predicted probability based on the logistic regression formula using coefficient values in Table 2, and ranges from 0% (lowest probability for early death after intubation) to 100% (highest probability for early death after intubation). The distribution of the ICOP score in the derivation and validation cohorts are shown in eFigure 2 in the Supplementary material.

**Figure 2:**
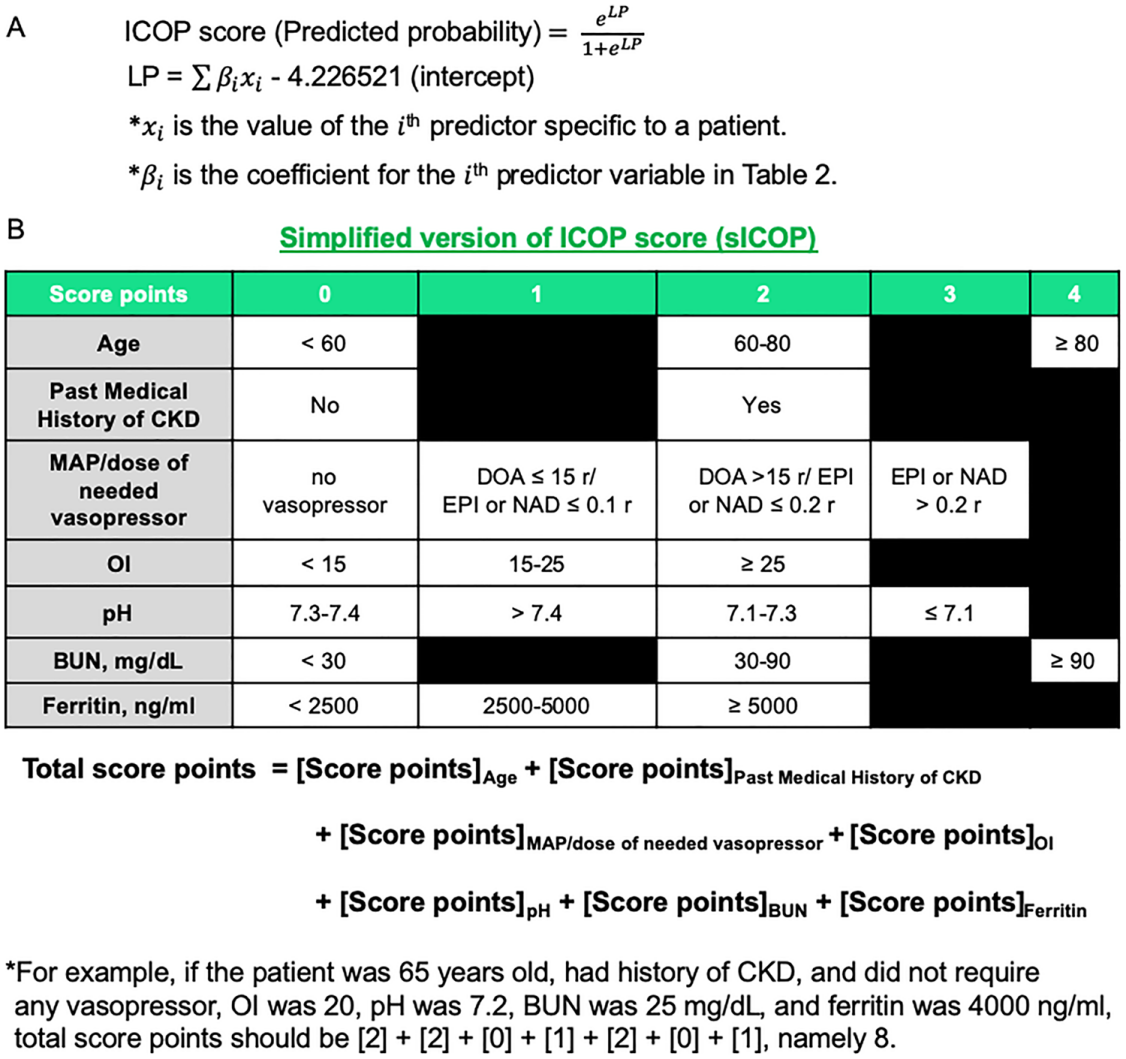
Formula of ICOP Score and sICOP. (**A**) The ICOP score is calculated as the predicted probability by using coefficient values in Table 2. (**B**) The formula of simplified version of ICOP (sICOP) score. The sICOP score was calculated by summing up each score points with the corresponding categorical variable. *CKD*, chronic kidney disease; *MAP*, mean arterial pressure; *OI*, oxygen index; *BUN*, blood urea nitrogen.

For clinical convenience, we abridged the ICOP score and developed the simplified version of the ICOP score (sICOP). All continuous variables (age, and the values of BUN, ferritin, and OI) were changed to categorical, and each odds ratio was rounded as shown in eTable 3 in the Supplementary material. The exact formulas to calculate the ICOP and sICOP scores are shown in Fig. 2A,B. An Excel spreadsheet allows calculation of ICOP score (Excel file available upon request), and the development of a web application for ICOP score is under consideration.

**Figure 3:**
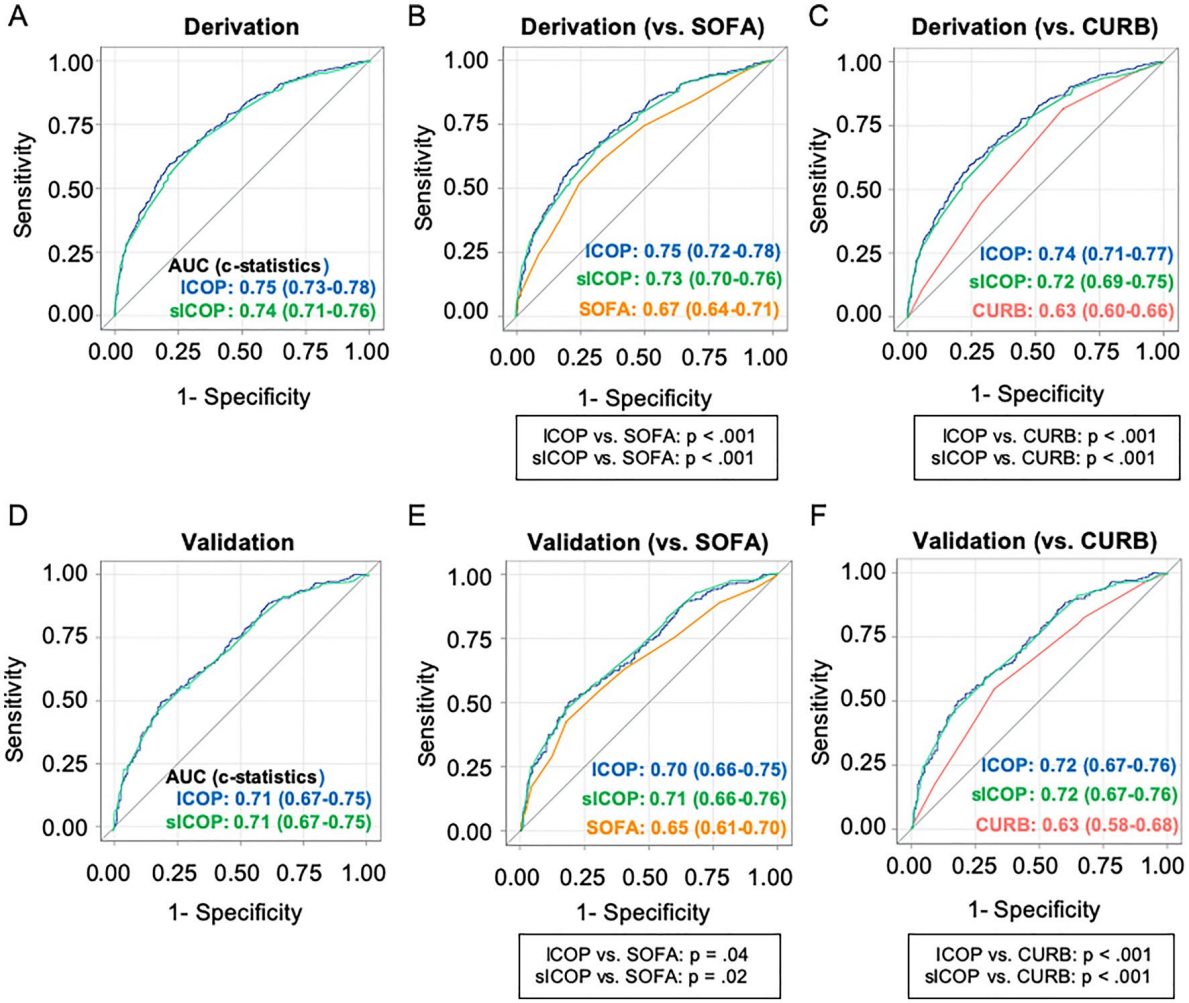
Receiver operating characteristic curves for 14-day mortality after intubation. The Receiver Operating Characteristic (ROC) Curves of our predictive score (ICOP score) and simplified version of the score (sICOP) for 14-day mortality after intubation in derivation (**A**) and validation cohorts (**D**). The *c*-statistics of ICOP score and sICOP were compared with SOFA score (**B**,**E**) and CURB-65 score (**C**,**F**) by DeLong’s test. In the comparison with SOFA score or CURB-65 score, only patients in whom all variables needed for the calculation of the corresponding scores were available, were analyzed (n = 1046 in derivation and n = 446 in validation cohorts for SOFA score, and n = 1158 in derivation and n = 514 in validation cohorts for CURB-65 score). *AUC*, area under the receiver operating characteristic curve; *SOFA*, sequential organ failure assessment score (SOFA score); *CURB*, CURB-65 score.

The *c*-statistic of the ICOP score was 0.75 (95% confidence interval [95% CI] 0.73–0.78) in the derivation cohort, 0.72 (0.68–0.76) in the internal validation and 0.71 (0.67–0.75) in the external validation. The *c*-statistic of sICOP was 0.74 (95%CI 0.71–0.76) in the derivation cohort, 0.73 (0.70–0.75) in the internal validation and 0.71 (0.67–0.75) in the external validation (Fig. 3A,D). We also compared the *c*-statistics of our scores separately with each of the existing predictive scores, SOFA and CURB-65 score by using only patients in whom these scores were evaluable (n = 1046 and n = 446 for SOFA score, and n = 1158 and n = 514 for CURB-65 score). These comparisons demonstrated that the *c*-statistic of the ICOP score was significantly greater than the corresponding values for SOFA and CURB-65 scores in the derivation cohort (vs. SOFA: 0.67 (0.64–0.71), *P* < 0.001, and vs. CURB-65: 0.63 (0.60–0.66), *P* < 0.001), as well as in the validation cohort (vs. SOFA: 0.65 (0.61–0.70), *P* = 0.04, and vs. CURB-65: 0.63 (0.58–0.68), *P* < 0.001). Similarly, the *c*-statistics for sICOP were also significantly greater than both SOFA and CURB-65 (Fig. 2B,C,E,F). We also evaluated the predictive accuracy of 28-day mortality after intubation as a secondary outcome. The AUCs of the ICOP and sICOP scores were also significantly greater than SOFA and CURB-65 scores (eFigure 3 in the Supplementary material).

As a sensitivity analysis to examine the model selection process, logistic regression using the Least Absolute Shrinkage Selection Operator (LASSO regression) was also performed on the derivation set. The best-tuned model yielded c = 0.74 (eFigure 4 in the Supplementary material), which was similar to the performance of the ICOP score. The LASSO model was less parsimonious, retaining all 36 candidate variables (eTable 4 in the Supplementary material).

**Figure 4:**
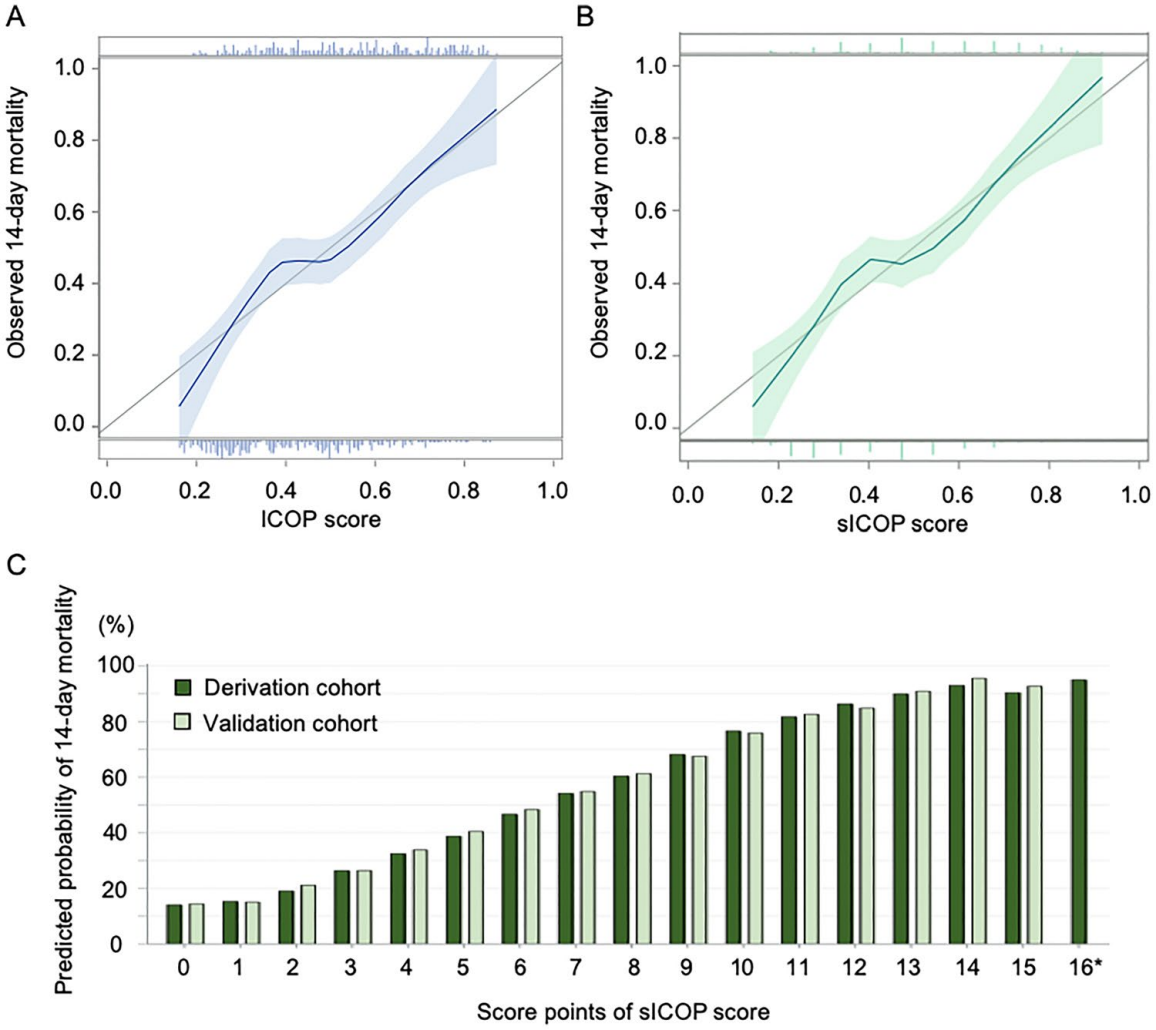
Calibration plots in validation cohort of ICOP score/sICOP and predicted probability of 14-day mortality by each point on sICOP. Calibration plots of our predictive scores (ICOP score and sICOP) for 14-day mortality after intubation (**A**,**B**). The calibration curves represent the relationship between the mortality predicted by the ICOP score (x-axis) and the observed mortality (y-axis). The gray lines in figures represent a perfect calibration. A calibration curve below the gray line indicates that the score overestimates the mortality. (**C**) Predicted probability of 14-days mortality after intubation by each score point on sICOP (0–16 points). *There were no patients who scored 16 points on sICOP in the validation set.

In order to examine the ability of the ICOP and sICOP scores to rank patients according to the risk, we evaluated the calibration of these scores. The calibration plots demonstrated good agreement between the observed and predicted probabilities (Fig. 4A,B). The cut off points for the predicted probabilities showing > 50% and > 80%, respectively, were 7 and 11 (Fig. 4C).

## Discussion

In our present, multicenter, retrospective study performed in New York, which saw the initial highest incidence rates of COVID-19 in the US^17^, we developed the ICOP score and simplified version of the score to predict the risk of early death of COVID-19 patients after intubation by using variables that can be easily obtained in the early phase after intubation. The AUCs of these scores in external validation were greater than 0.7, which is regarded as an acceptable predictive accuracy^18,19^. In actual clinical practice, we believe that risk stratification based on the ICOP score is mainly helpful for guiding joint decision-making among patients’ family and medical staff in regard to future treatment options, distinguishing patients who can benefit from the valuable and limited medical resources, and determining when to advance intubated patients onto ECMO.

Reallocating and distributing the valuable and limited medical and human resources available have been one of the biggest concerns during this COVID-19 pandemic. The lack of resources was remarkably observed in Italy^20^, and also, in New York, where many patients were treated in “temporary” ICUs because of the limited capacity of traditional ICUs, despite patients requiring intubation^6^. Under these circumstances, the ICOP score may be helpful because we can potentially identify patients who are so severely ill and require intensive care treatment. If certain patients have a higher probability of early death based on our ICOP score, they should be treated preferentially in a “traditional” ICU, rather than a “temporary” ICU.

Also, the ICOP score may be useful for detecting patients who potentially require respiratory ECMO. Several studies have suggested that respiratory ECMO in intubated patients with COVID-19 has the potential for improving their outcomes as seen in other respiratory infectious diseases^13,21–23^. Since Extracorporeal Life Support Organization (ELSO) guidelines recommend the consideration or direct application of respiratory ECMO depending on the predicted patient mortality of 50% or 80%, respectively^24^, COVID-19 patients with an even greater mortality risk based on our ICOP score should be considered as candidates for ECMO. If they are in a hospital where ECMO is not available, transportation to the tertiary hospitals as soon as possible should be considered.

There were 7 variables selected for our predictive score that were independently associated with early death after intubation: age, history of chronic kidney disease, the values of mean arterial pressure or dose of needed vasopressors, blood urea nitrogen, ferritin, OI, and pH. Some of these selected variables can serve as surrogates for the functions of essential organs, which are also used for existing predictive scores such as the SOFA and APACHE II scores^25,26^. Furthermore, some variables such as age and ferritin have been already shown to be associated with increased mortality of COVID-19 patients in recent studies^8,27,28^. Among them, ferritin is known to be associated with the development of the inflammatory cytokine storm, and given that cytokine storm is a major cause of death of COVID-19 patients^29^, it is also reasonable to include ferritin in our predictive score. The greatest strength of the ICOP score is the specificity of its use for COVID-19 patients, while other more general predicted scores lose some of their predictive power in the case of this new, severe viral illness. In fact, the predictive accuracy of our score was statistically significantly greater than these other existing scores, such as SOFA score and CURB-65 score, both of which did not show acceptable predictive accuracy for our analyzed patients.

Among the variety of statistical approaches used for the development of predictive scores, we selected a logistic regression model using backward selection^30,31^. One of the biggest difficulties of conducting research for COVID-19 is that the pathophysiology of COVID-19 has not been completely understood and there has been minimal evidence for the variables that are associated with the mortality of intubated COVID-19 patients. In this context, our score was developed using a more understandable approach for clinicians, who may not likely use it in actual clinical practice otherwise. Secondly, a simplified score (sICOP score) was also developed for practicality, without the need for any electronic devices for its calculation. Many previous clinical studies have used these approaches to develop simplified scores^32,33^. Other alternatives, such as LASSO, may significantly improve the predictive accuracy compared with the conventional approach; however, our sensitivity analysis showed that the predictive accuracy with LASSO was similar to our approach in this study, supporting the predictive capability of our score.

In this study, patients admitted to 12 Northwell hospitals in the greater New York City area were geographically divided into derivation and validation cohorts. It is possible that by using this split, selection bias was introduced. However, nonrandom splits by location are considered a stronger design to evaluate the transportability of a model and recommended over the split-sample approach in the Tripod statement^14^. A broader external validation in the future is necessary for more information on the utility of this score. Also, patients who were transferred from outside of Northwell Health after intubation or who transferred to outside of Northwell Health within 14 days were excluded because any information needed for the calculation of the score or the information of the outcome was not available. There is a possibility that the overall results might potentially change if we could include them, but we believe this effect is minimal because only 5 and 42 among 2182 patients (0.2% and 2%) were excluded by each reason (transferred from outside of Northwell and transferred to outside of Northwell within 14 days), respectively. In fact, we confirmed that if we treated the patients who transferred to outside of Northwell Health within 14 days as either having the event or as event-free, the predictive accuracies of the final models were similar (data not shown). Also, we categorized all continuous variables in the sICOP score in order to create a simple score that is easy to calculate. The categorization of continuous variables will likely decrease model performance. Furthermore, predictive scores should be carefully considered since they only indicate the general probability of an outcome in the general population without offering the precise probability in individual patients–that is, the results of mortality prediction for individual patients using the score are not absolute^34^. The final therapeutic strategy should not be solely based on the predictive score, but also involve a variety of different factors. As the numbers of cases continue to rise and many new areas are beginning to experience the same devastation that New York recently overcame, the use of our ICOP score for determining early morality of intubated COVID-19 patients may help to alleviate some of the burden of this disease faced by the medical staff, healthcare systems, and families of those suffering.

## Conclusions

Among intubated COVID-19 patients, there are 7 predictors of early mortality after intubation. The externally validated predictive score may help clinicians estimate early mortality risk after intubation and provide guidance for deciding the most effective patient therapies.

## Supplementary Information

Supplementary Information.

## Data Availability

The datasets used and analyzed during the current study are available from the corresponding author upon reasonable request.
