# Supplementary material for: Intubated COVID-19 predictive (ICOP) score for early mortality after intubation in patients with COVID-19
Source: Sci Rep. 2021 Oct 26;11:21124. doi: 10.1038/s41598-021-00591-1 (PMC8548515; doi:10.1038/s41598-021-00591-1)

**[Supplementary material]**

**Intubated COVID-19 Predictive (ICOP) Score for Early Mortality After Intubation in Patients With COVID-19**

Mitsuaki Nishikimi, MD; Rehana Rasul, MA, MPH; Cristina P. Sison, PhD; Daniel Jafari, MD, MPH; Muhammad Shoaib, BA; Koichiro Shinozaki, MD, PhD; Timmy Li, PhD; Kei Hayashida, MD, PhD; Daniel M. Rolston, MD, MSHPM; Jamie S. Hirsch, MD, MA, MSB; Lance B. Becker, MD, FAHA; and the Northwell Health COVID-19 Research Consortium

The Northwell Health COVID-19 Research Consortium Authors: Matthew A. Barish, MD; Douglas P. Barnaby, MD, MSc; Santiago J. Miyara, MD; Edith Burns, MD; Stuart L. Cohen, MD; Jennifer Cookingham, MHA; Andrew J. Dominello, BA^3^; Jennifer C. Johnson, MS, MA; Zachary M. Kozel, MD; Brian Lima, MD; Ariana K. McGinn, BA^3^; Ernesto P. Molmenti, MD, PhD, MBA, FACS; Rachel Monane, BBA; and Marc d. Paradis, SM

**eMethod**

**Participating Hospital**

Our study used the data of patients in 12 hospitals affiliated to Northwell Health, New York. Among these 12 hospitals, Long Island Jewish Forest Hills, Lenox Hill Hospital, Long Island Jewish Hospital, Staten Island University Hospital North, and Staten Island University Hospital South are in New York City, Huntington Hospital and Southside Hospital are in Suffolk Country, and Glen Cove Hospital, Long Island Jewish Valley Stream, North Shore University Hospital, Plainview Hospital, and Syosset Hospital are in Nassau County.

**Data Extraction**

Samples for obtaining information on the results of laboratory tests were collected within a time window from 72 hours prior to intubation until 24 hours after intubation. Information on the vital signs at the time of intubation was obtained within the time window of 1 hour from the time of intubation. If there were more than two records available for either the laboratory test results or vital signs at the time of intubation, the values measured at the time-point closest to the intubation were selected. Initial arterial blood gas analysis results were defined as the first test results available after intubation. If the first test was later than 24 hours after intubation, we excluded these values and counted them as missing values. The values of NLR, PF ratio, and AaDO_2_ were calculated by neutrophil count and lymphocyte count, FiO_2_ and PaO_2_, and PaO_2_, PaCO_2_ and FiO_2_, respectively, all of which were directly extracted from the electronic medical chart. Oxygen index (OI) was calculated by using the variables of mean airway pressure and PF ratio.^1^

**Sample Size**

The sample size was based on the number of patients in the COVID-19 registry that met inclusion criteria from March 2, 2020 to April 27, 2020. The derivation and validation cohorts were based on geographic location. There were 608 events in the derivation set and 267 events in the validation cohort, meeting minimum requirements for developing and validating a prognostic model.^2,3^

**Categorizing and Collinearity**

Before modeling, we categorized several variables which are physiologically regarded as nonlinearly related with mortality (body mass index [BMI], mean arterial pressure [MAP]/ dose of need vasopressors, heart rate, urinary output, albumin, sodium, potassium, white blood cell (WBC) count, hematocrit, pH, PaCO_2_, HCO_3_, creatinine, and neutrophil-to-lymphocyte ratio (NLR) based on some references.^4-11^ As for other candidates, we checked for linearity relationships. Variables that showed linearity (age, alkaline phosphate [ALP], total bilirubin, total protein, blood urea nitrogen [BUN], c-reactive protein [CRP], ferritin, lactate, red cell distribution width [RCDW], oxygen index [OI], AaDO_2_, and PF ratio) were regarded as continuous variables. All remaining variables (D-dimer, procalcitonin and platelet count) were categorized based on some references.^5,12,13^ Collinearity was concluded if variance inflation factor ≥5. As a result, PF ratio, which showed collinearity with OI, and PaCO_2_, which showed collinearity with pH, were removed as candidate predictors.

**Missing Data and Data Imputations**

In the derivation cohort, all candidate predictor variables had <25% missing values (eTable 1). MAP/ dose of need vasopressors (12.2%), procalcitonin (16.0%), urinary output (21.8%), CRP (11.0%), D-dimer (24.4%), and ferritin (15.0%) had >10% missing values. Missing data was more frequent among patients who were severe, which was indicated by number of comorbidities and mortality status. Additionally, missing data varied by hospital and week of the epidemic. It is recommended that imputation is performed for prediction models instead of a complete-case analysis in the presence of missing data, assuming data are missing at random (MAR). Given that the patterns found suggested that data were MAR, conditional on other factors collected, the method of multiple imputation was chosen. The derivation cohort was imputed 33 times. The number of imputations was based on the formula by von Hippel.^14^ Urinary output had the highest fraction of missing information (FMI), 40%, and accepting a change in standard error (SE) over imputed datasets of 0.05, 33 imputations were needed. Imputed values were generated by predictive mean matching for continuous variables and discriminant analysis for categorical variables. Mortality, week, and hospital were used as auxiliary variables. In the validation cohort, multiple imputation was performed 33 times using the same methods.

**Calculation of the optimism corrected c-statistic**

Internal validation was assessed using the optimism corrected c-statistic^15^. First, 100 bootstrapped samples with replacement of size 1389 were generated. Second, for each bootstrapped sample, 33 imputed datasets were generated. Logistic regression with backwards selection was performed on each imputed dataset using all candidate variables. Variables appearing > 50% of the models from each imputed dataset per bootstrapped sample were used to fit a new model on those imputed datasets. Results were pooled to determine the final model per bootstrapped sample. Third, each model was used to score imputed datasets per bootstrapped sample and the average c-statistic across imputed datasets for that bootstrapped sample (c_boot_). Fourth, each model was also used to score the original set of 33 imputed datasets of the derivation sample and average c-statistics were calculated (c_orig(m)_). Fifth, the difference between c_boot_ and c_orig(m)_ was calculated and averaged to determine optimism. The optimism-corrected c-statistic was then determined by subtracting the optimism from the apparent c-statistic.

**Sensitivity Analysis using LASSO Regression**

As a sensitivity analysis to examine the model selection process, logistic regression using the Least Absolute Shrinkage Selection Operator (LASSO regression) was also performed on the derivation set. The best-tuned model based on the AUC after three repetitions of 10-fold cross validation was found and its c-statistic was compared to that of the ICOP score. Analyses were performed using the Caret (https://CRAN.R-project.org/package=caret) and glmnet (https://www.jstatsoft.org/v33/i01/.) packages in R (The R Foundation for Statistical Computing, Vienna, Austria). Prior to fitting the models, the derivation set was imputed once using the package missRanger (https://github.com/mayer79/missRanger).

**Supplemental References**

1. DesPrez K, McNeil JB, Wang C, Bastarache JA, Shaver CM, Ware LB. Oxygenation Saturation Index Predicts Clinical Outcomes in ARDS. *Chest.* 2017;152(6):1151-1158.

2. Collins GS, de Groot JA, Dutton S, et al. External validation of multivariable prediction models: a systematic review of methodological conduct and reporting. *BMC Med Res Methodol.* 2014;14:40.

3. Steyerberg EW. Validation in prediction research: the waste by data splitting. *J Clin Epidemiol.* 2018;103:131-133.

4. Obesity: preventing and managing the global epidemic. Report of a WHO consultation. *World Health Organ Tech Rep Ser.* 2000;894:i-xii, 1-253.

5. Vincent JL, Moreno R, Takala J, et al. The SOFA (Sepsis-related Organ Failure Assessment) score to describe organ dysfunction/failure. On behalf of the Working Group on Sepsis-Related Problems of the European Society of Intensive Care Medicine. *Intensive Care Med.* 1996;22(7):707-710.

6. Bellomo R, Ronco C, Kellum JA, Mehta RL, Palevsky P, Acute Dialysis Quality Initiative w. Acute renal failure - definition, outcome measures, animal models, fluid therapy and information technology needs: the Second International Consensus Conference of the Acute Dialysis Quality Initiative (ADQI) Group. *Crit Care.* 2004;8(4):R204-212.

7. Cholongitas E, Papatheodoridis GV, Vangeli M, Terreni N, Patch D, Burroughs AK. Systematic review: The model for end-stage liver disease--should it replace Child-Pugh's classification for assessing prognosis in cirrhosis? *Aliment Pharmacol Ther.* 2005;22(11-12):1079-1089.

8. Knaus WA, Draper EA, Wagner DP, Zimmerman JE. APACHE II: a severity of disease classification system. *Crit Care Med.* 1985;13(10):818-829.

9. Kaukonen KM, Bailey M, Pilcher D, Cooper DJ, Bellomo R. Systemic inflammatory response syndrome criteria in defining severe sepsis. *N Engl J Med.* 2015;372(17):1629-1638.

10. Shekar K, Badulak J, Peek G, et al. Extracorporeal Life Support Organization Coronavirus Disease 2019 Interim Guidelines: A Consensus Document from an International Group of Interdisciplinary Extracorporeal Membrane Oxygenation Providers. *ASAIO J.* 2020;66(7):707-721.

11. Zimmerman JE, Wagner DP, Draper EA, Wright L, Alzola C, Knaus WA. Evaluation of acute physiology and chronic health evaluation III predictions of hospital mortality in an independent database. *Crit Care Med.* 1998;26(8):1317-1326.

12. Harbarth S, Holeckova K, Froidevaux C, et al. Diagnostic value of procalcitonin, interleukin-6, and interleukin-8 in critically ill patients admitted with suspected sepsis. *Am J Respir Crit Care Med.* 2001;164(3):396-402.

13. Bakhtiari K, Meijers JC, de Jonge E, Levi M. Prospective validation of the International Society of Thrombosis and Haemostasis scoring system for disseminated intravascular coagulation. *Crit Care Med.* 2004;32(12):2416-2421.

14. Hippel PT. How many imputations do you need?

A two‐stage calculation using a quadratic rule. *Sociological Methods and Research.* 2018;49(3):699-718.

15. Harrell FE, Jr., Lee KL, Mark DB. Multivariable prognostic models: issues in developing models, evaluating assumptions and adequacy, and measuring and reducing errors. *Stat Med.* 1996;15(4):361-387.

**eTables**

**eTable 1. Percentage of Missing Values for Variables.**

|  | **% Missing**  **Derivation cohort**  **(n = 1389)** | **% Missing**  **Validation cohort**  **(n = 556)** |
| --- | --- | --- |
| **Demographics** |  |  |
| Age | 0 | 0 |
| Gender | 0 | 0 |
| BMI | 6.6 | 1.6 |
| **Comorbidities** |  |  |
| Hypertension | 0 | 0 |
| Diabetes | 0 | 0 |
| Heart Disease | 0 | 0 |
| Lung Disease | 0 | 0 |
| Cancer | 0 | 0 |
| Dementia | 0 | 0 |
| CKD | 0 | 0 |
| Chronic Liver Diseases | 0 | 0 |
| **Vital signs** |  |  |
| Heart Rate | 3.7 | 0.9 |
| MAP/ dose of needed vasopressor | 12.2 | 5.6 |
| Urine Output | 21.7 | 18.5 |
| **Laboratory values** |  |  |
| Albumin | 1.2 | 1.1 |
| ALP | 1.2 | 1.1 |
| Total Bil | 1.2 | 1.1 |
| Total protein | 1.4 | 1.3 |
| BUN | 0.2 | 0 |
| Creatinine | 0.2 | 0 |
| CRP | 11.0 | 7.2 |
| D-dimer | 24.4 | 13.3 |
| Ferritin | 15.0 | 7.9 |
| Hematocrit | 0.3 | 0 |
| NLR | 3.7 | 6.7 |
| Plat Count | 0.4 | 0 |
| Potassium | 0.4 | 0 |
| Procalcitonin | 15.9 | 15.5 |
| RCDW | 0.3 | 0.2 |
| Sodium | 0.2 | 0 |
| WBC Count | 0.3 | 0 |
| Lactate | 9.1 | 13 |
| **Blood Gases** |  |  |
| PF ratio | 2.7 | 1.1 |
| PaCO_2_ | 2.5 | 3.1 |
| AaDO_2_ | 4.1 | 4.1 |
| Oxygen Index | 12.1 | 3.1 |
| HC0_3_ | 2.5 | 9.5 |
| pH | 3.7 | 6.1 |

Abbreviations: IQR, interquartile range; BMI, body mass index; CKD, chronic kidney disease; MAP, mean arterial pressure; ALP, alkaline phosphatase; Bil, bilirubin; BUN, blood urea nitrogen; CRP, C-reactive protein; NLR, neutrophil to lymphocyte ratio; Plat, platelet; RCDW, red blood cell distribution width; WBC, white blood cell.

**eTable 2. Variable Candidates for the Predictive Score**

|  | **Derivation cohort (n=1389)** | | | **Validation cohort (n=556)** | | |
| --- | --- | --- | --- | --- | --- | --- |
|  | **14-days mortality** | |  | **14-days mortality** | |  |
|  | **Yes** | **No** | **Total** | **Yes** | **No** | **Total** |
| **Demographics** |  |  |  |  |  |  |
| Age, median (IQR), yrs | 69 (60-77) | 62 (53-72) | 65 (56-73) | 69 (61-77) | 64 (56-73) | 67 (58-75) |
| Gender, male, n (%) | 444 (73.0) | 520 (66.6) | 964 (69.4) | 183 (68.5) | 183 (63.3) | 366 (65.8) |
| BMI, *n* (%) |  |  |  |  |  |  |
| <25 | 124 (22.2) | 121 (16.4) | 245 (18.9) | 60 (22.9) | 56 (19.7) | 116 (21.2) |
| ≥25 to <30 | 189 (33.9) | 254 (34.4) | 443 (34.2) | 98 (37.4) | 98 (34.4) | 196 (35.8) |
| ≥30 to <40 | 202 (36.2) | 292 (39.5) | 494 (38.1) | 82 (31.3) | 103 (36.1) | 185 (33.8) |
| ≥40 | 43 (7.7) | 72 (9.7) | 115 (8.9) | 22 (8.4) | 28 (9.8) | 50 (9.1) |
| **Comorbidities,** n (%) |  |  |  |  |  |  |
| Hypertension | 401 (66.0) | 448 (57.4) | 849 (61.1) | 175 (65.5) | 171 (59.2) | 346 (62.2) |
| Diabetes | 270 (44.4) | 294 (37.6) | 564 (40.6) | 124 (46.4) | 120 (41.5) | 244 (43.9) |
| Heart Disease | 225 (37.0) | 160 (20.5) | 385 (27.7) | 93 (34.8) | 80 (27.7) | 173 (31.1) |
| Lung Disease | 125 (20.6) | 112 (14.3) | 237 (17.1) | 46 (17.2) | 53 (18.3) | 99 (17.8) |
| Cancer | 71 (11.7) | 60 (7.7) | 131 (9.4) | 37 (13.9) | 28 (9.7) | 65 (11.7) |
| Dementia | 28 (4.6) | 26 (3.3) | 54 (3.9) | 13 (4.9) | 6 (2.1) | 19 (3.4) |
| CKD | 83 (13.7) | 37 (4.7) | 120 (8.6) | 29 (10.9) | 17 (5.9) | 46 (8.3) |
| Chronic Liver Diseases | 11 (1.8) | 16 (2.1) | 27 (1.9) | 9 (3.4) | 5 (1.7) | 14 (2.5) |
| **Vital signs** |  |  |  |  |  |  |
| Heart Rate, n (%), beats/min |  |  |  |  |  |  |
| <70 | 34 (5.8) | 37 (4.9) | 71 (5.3) | 11 (4.2) | 10 (3.5) | 21 (3.8) |
| ≥70 to <90 | 100 (17.1) | 167 (22.2) | 267 (20.0) | 38 (14.3) | 48 (16.8) | 86 (15.6) |
| ≥90 to <110 | 177 (30.2) | 214 (28.5) | 391 (29.2) | 75 (28.3) | 84 (29.4) | 159 (28.9) |
| ≥110 to <130 | 171 (29.2) | 223 (29.7) | 394 (29.5) | 82 (30.9) | 82 (28.7) | 164 (29.8) |
| ≥130 | 104 (17.8) | 110 (14.7) | 214 (16.0) | 59 (22.3) | 62 (21.7) | 121 (22.0) |
| MAP/ dose of needed vasopressor, n (%) |  |  |  |  |  |  |
| MAP ≥70 | 193 (35.7) | 340 (50.0) | 533 (43.7) | 141 (52.0) | 89 (35.0) | 230 (43.8) |
| MAP <70, no vasopressor | 92 (17.0) | 134 (19.7) | 226 (18.5) | 56 (20.1) | 40 (15.8) | 96 (18.3) |
| DOA ≤15r/ EPI or NAD ≤0.1 r | 94 (17.4) | 118 (17.4) | 212 (17.4) | 41 (15.1) | 39 (15.4) | 80 (15.2) |
| DOA >15 r /EPI or NAD ≤0.2 r | 58 (10.7) | 48 (7.1) | 106 (8.7) | 16 (5.9) | 28 (11.0) | 44 (8.4) |
| EPI or NAD >0.2 r | 103 (19.1) | 40 (5.9) | 143 (11.7) | 17 (6.3) | 58 (22.8) | 75 (14.3) |
| Urine Output, n (%), ml/kg/h |  |  |  |  |  |  |
| 0 | 47 (9.9) | 22 (3.6) | 69 (6.3) | 20 (9.4) | 8 (3.3) | 28 (6.2) |
| >0 to ≤0.3 | 26 (5.5) | 13 (2.1) | 39 (3.6) | 15 (7.0) | 7 (2.9) | (4.9) |
| >0.3 to ≤0.5 | 16 (3.4) | 7 (1.1) | 23 (2.1) | 8 (3.8) | 6 (2.5) | 14 (3.1) |
| > 0.5 to ≤1.0 | 40 (8.4) | 25 (4.1) | 65 (6.0) | 17 (8.0) | 12 (5.0) | 29 (6.4) |
| >1.0 | 345 (72.8) | 547 (89.1) | 892 (82.0) | 153 (71.8) | 207 (86.3) | 360 (79.5) |
| **Laboratory values** |  |  |  |  |  |  |
| Albumin, n (%), g/dL |  |  |  |  |  |  |
| <2.8 | 245 (40.8) | 272 (35.2) | 517 (37.7) | 151 (57.0) | 146 (51.2) | 297 (54.0) |
| ≥2.8 to <3.5 | 277 (46.2) | 372 (48.2) | 649 (47.3) | 90 (34.0) | 101 (35.4) | 191 (34.7) |
| ≥3.5 | 78 (13.0) | 128 (16.6) | 206 (15.0) | 24 (9.1) | 38 (13.3) | 62 (11.3) |
| ALP, median (IQR), U/L | 92 (66-133) | 84 (63-117) | 87 (64-123) | 96 (67-157) | 89 (61-127) | 93 (64-138) |
| Total Bil, median (IQR), mg/dL | 0.6 (0.4-0.9) | 0.5 (0.4-0.8) | 0.6 (0.4-0.9) | 0.6 (0.4-0.8) | 0.6 (0.4-0.7) | 0.6 (0.4-0.8) |
| Total protein, median (IQR), g/dL | 6.8 (6.2-7.4) | 6.8 (6.2-7.3) | 6.8 (6.2-7.4) | 7 (6.25-7.5) | 7 (6.4-7.6) | 7 (6.4-7.6) |
| BUN, median (IQR), mg/dL | 33 (22-55) | 21 (14-34) | 26 (16-43) | 35 (21-54) | 23 (16-36) | 27 (17-44) |
| Creatinine, n (%), mg/dL |  |  |  |  |  |  |
| <1.2 | 219 (36.1) | 501 (64.3) | 720 (52.0) | 108 (40.5) | 187 (64.7) | 295 (53.1) |
| ≥1.2 to <2 | 191 (31.5) | 159 (20.4) | 350 (25.3) | 69 (25.8) | 53 (18.3) | 122 (21.9) |
| ≥2 to <3.5 | 103 (17.0) | 78 (10.0) | 181 (13.1) | 53 (19.9) | 28 (9.7) | 81 (14.6) |
| ≥3.5 to <5 | 35 (5.8) | 13 (1.7) | 48 (3.5) | 11 (4.1) | 8 (2.8) | 19 (3.4) |
| ≥5 | 59 (9.7) | 28 (3.6) | 87 (6.3) | 26 (9.7) | 13 (4.5) | 39 (7.0) |
| CRP, median (IQR), mg/L | 16 (8-26) | 15 (8-25) | 16 (8-25) | 13 (6-25) | 15 (8-22) | 14 (7-24) |
| D-dimer, n (%) |  |  |  |  |  |  |
| <5 times the upper limit of normal | 197 (43.8) | 308 (51.3) | 505 (48.1) | 106 (45.5) | 125 (50.2) | 231 (47.9) |
| ≥5 times to <10 times | 53 (11.8) | 79 (13.2) | 132 (12.6) | 23 (9.9) | 26 (10.4) | 49 (10.2) |
| ≥10 times to <25 times | 89 (19.8) | 96 (16.0) | 185 (17.6) | 52 (22.3) | 50 (20.1) | 102 (21.2) |
| ≥25 times to <50 times | 40 (8.9) | 59 (9.8) | 99 (9.4) | 16 (6.9) | 21 (8.4) | 37 (7.7) |
| ≥50 times | 71 (15.8) | 58 (9.7) | 129 (12.3) | 36 (15.5) | 27 (10.8) | 63 (13.1) |
| Ferritin, median (IQR), x10^3^ ng/ml | 1.4 (0.7-2.5) | 1.0 (0.6-1.8) | 1.1 (0.7-2.1) | 1.3 (0.8-2.6) | 1.0 (0.6-2.0) | 1.1 (0.7-2.3) |
| Hematocrit, n (%) |  |  |  |  |  |  |
| <30 | 70 (11.5) | 68 (8.7) | 138 (10.0) | 29 (10.9) | 32 (11.1) | 61 (11.0) |
| ≥30 to <46 | 455 (75.0) | 631 (81.1) | 1086 (78.4) | 194 (72.7) | 217 (75.1) | 411 (73.9) |
| ≥46 | 82 (13.5) | 79 (10.2) | 161 (11.6) | 44 (16.5) | 40 (13.8) | 84 (15.1) |
| NLR, n (%) |  |  |  |  |  |  |
| <10 | 182 (32.3) | 292 (41.7) | 474 (37.5) | 73 (34.4) | 104 (41.8) | 177 (38.4) |
| ≥10 to <20 | 197 (35.0) | 245 (35.0) | 442 (35.0) | 75 (35.4) | 89 (35.7) | 164 (35.6) |
| ≥20 to <30 | 76 (13.5) | 75 (10.7) | 151 (11.95) | 27 (12.7) | 29 (11.7) | 56 (12.2) |
| ≥30 to <40 | 48 (8.5) | 33 (4.7) | 81 (6.4) | 14 (6.6) | 15 (6.0) | 29 (6.3) |
| ≥40 | 60 (10.7) | 56 (8.0) | 116 (9.2) | 23 (10.9) | 12 (4.8) | 35 (7.6) |
| Plat Count, n (%), 10^5/^/uL |  |  |  |  |  |  |
| <1 | 23 (3.8) | 24 (3.1) | 47 (3.4) | 8 (3) | 9 (3.11) | 17 (3.06) |
| ≥1 to <1.5 | 79 (13.0) | 71 (9.2) | 150 (10.9) | 40 (15.0) | 32 (11.1) | 72 (13.0) |
| ≥1.5 | 505 (83.2) | 681 (87.8) | 1186 (85.8) | 219 (82.0) | 248 (85.8) | 467 (84.0) |
| Potassium, n (%), mmol/L |  |  |  |  |  |  |
| <3.5 | 45 (7.5) | 65 (8.3) | 110 (8.0) | 24 (9.0) | 23 (8.0) | 47 (8.5) |
| ≥3.5 to <6 | 520 (86.1) | 700 (89.9) | 1220 (88.2) | 226 (84.6) | 256 (88.6) | 482 (86.7) |
| ≥6 | 39 (6.5) | 14 (1.8) | 53 (3.8) | 17 (6.4) | 10 (3.5) | 27 (4.9) |
| Procalcitonin, n (%), ng/mL |  |  |  |  |  |  |
| <0.5 | 201 (40.0) | 355 (53.3) | 556 (47.6) | 91 (41.2) | 134 (53.8) | 225 (47.9) |
| ≥0.5 to <2 | 176 (35.1) | 198 (29.7) | 374 (32.0) | 83 (37.6) | 64 (25.7) | 147 (31.3) |
| ≥2 to <10 | 81 (16.1) | 70 (10.5) | 151 (12.9) | 33 (14.9) | 37 (14.9) | 70 (14.9) |
| ≥10 | 44 (8.8) | 43 (6.5) | 87 (7.5) | 14 (6.3) | 14 (5.6) | 28 (6.0) |
| RCDW, median (IQR), % | 14 (13-15) | 14 (13-15) | 14 (13-15) | 14 (13-16) | 14 (13-15) | 14 (13-15) |
| Sodium, n (%), mmol/L |  |  |  |  |  |  |
| <130 | 34 (5.6) | 56 (7.2) | 90 (6.5) | 14 (5.2) | 22 (7.6) | 36 (6.5) |
| ≥130 to <150 | 526 (86.7) | 699 (89.7) | 1225 (88.4) | 239 (89.5) | 257 (88.9) | 496 (89.2) |
| ≥150 | 47 (7.7) | 24 (3.1) | 71 (5.1) | 14 (5.2) | 10 (3.5) | 24 (4.3) |
| WBC Count, n (%), K/uL |  |  |  |  |  |  |
| <4 | 16 (2.6) | 12 (1.5) | 28 (2.0) | 7 (2.6) | 6 (2.1) | 13 (2.3) |
| ≥4 to <12 | 282 (46.5) | 391 (50.3) | 673 (48.6) | 106 (39.7) | 152 (52.6) | 258 (46.4) |
| ≥12 to <20 | 198 (32.6) | 256 (32.9) | 454 (32.8) | 85 (31.8) | 84 (29.1) | 169 (30.4) |
| ≥20 to <30 | 91 (15.0) | 96 (12.3) | 187 (13.5) | 55 (20.6) | 32 (11.1) | 87 (15.7) |
| ≥30 | 20 (3.3) | 23 (3.0) | 43 (3.1) | 14 (5.2) | 15 (5.2) | 29 (5.2) |
| Lactate, median (IQR),  mmol/L | 1.9 (1.3-3.2) | 1.6 (1.1-2.3) | 1.7 (1.2-2.6) | 2.2 (1.4-3.6) | 1.9 (1.4-3) | 2 (1.4-3.2) |
| **Blood Gases** |  |  |  |  |  |  |
| AaDO2, median (IQR) | 490  (385-560) | 482  (361-551) | 486.5  (375-554) | 511  (403-553) | 500  (400-556) | 507  (402-554) |
| Oxygen Index, median (IQR) | 14 (9-21) | 13 (8-20) | 13 (9-21) | 15 (10-21) | 14 (9-22) | 15 (9-21) |
| HC03, n (%), mmol/L |  |  |  |  |  |  |
| <15 | 35 (5.9) | 14 (1.8) | 49 (3.6) | 12 (5.0) | 10 (3.8) | 22 (4.4) |
| ≥15 to <18 | 52 (8.8) | 39 (5.1) | 91 (6.7) | 15 (6.3) | 9 (3.4) | 24 (4.8) |
| ≥18 to <22 | 159 (26.9) | 181 (23.7) | 340 (25.1) | 45 (18.9) | 41 (15.5) | 86 (17.1) |
| ≥22 to <32 | 327 (55.3) | 489 (64.1) | 816 (60.3) | 150 (63.0) | 186 (70.2) | 336 (66.8) |
| ≥32 | 18 (3.1) | 40 (5.2) | 58 (4.3) | 16 (6.7) | 19 (7.2) | 35 (7.0) |
| pH, n (%) |  |  |  |  |  |  |
| <7.10 | 62 (10.7) | 37 (4.9) | 99 (7.4) | 41 (16.2) | 26 (9.7) | 67 (12.8) |
| ≥7.10 to <7.20 | 91 (15.6) | 78 (10.3) | 169 (12.6) | 49 (19.4) | 29 (10.8) | 78 (14.9) |
| ≥7.20 to <7.30 | 168 (28.9) | 190 (25.2) | 358 (26.8) | 77 (30.4) | 75 (27.9) | 152 (29.1) |
| ≥7.30 to <7.40 | 173 (29.7) | 306 (40.5) | 479 (35.8) | 71 (28.1) | 107 (39.8) | 178 (34.1) |
| ≥ 7.40 | 88 (15.1) | 144 (19.1) | 232 (17.4) | 15 (5.9) | 32 (11.9) | 47 (9.0) |

Abbreviations: IQR, interquartile range; BMI, body mass index; CKD, chronic kidney disease; MAP, mean arterial pressure; r, mg/kg/min; DOA, dopamine; EPI, epinephrine; NAD, norepinephrine; ALP, alkaline phosphatase; Bil, bilirubin; BUN, blood urea nitrogen; CRP, C-reactive protein; NLR, neutrophil to lymphocyte ratio; Plat, platelet; RCDW, red blood cell distribution width; WBC, white blood cell.

**eTable 3. Simplified Coefficients for the Development of sICOP**

| **Predictor** | **Coef** | **OR (95% CI)** | ***P* value** | **Simplified**  **Coef** |
| --- | --- | --- | --- | --- |
| Age, y (Ref = < 60) |  |  |  |  |
| ≥ 60 to < 70 | 0.66 | 1.93 (1.43-2.62) | <.001 | **2** |
| ≥ 70 to < 80 | 0.77 | 2.16 (1.57-2.98) | <.001 | **2** |
| ≥ 80 | 1.46 | 4.32 (2.84-6.57) | <.001 | **4** |
| Past medical history of CKD | 0.63 | 1.88 (1.19-2.95) | .007 | **2** |
| BUN, mg/dL (Ref = < 30) |  |  |  |  |
| ≥ 30 to < 60 | 0.61 | 1.85 (1.40-2.43) | <.001 | **2** |
| ≥ 60 to < 90 | 0.66 | 1.93 (1.23-3.01) | .004 | **2** |
| ≥ 90 | 1.33 | 3.79 (2.01-7.15) | <.001 | **4** |
| Ferritin, ng/ml (Ref = < 2500) |  |  |  |  |
| ≥ 2500 to < 5000 | 0.31 | 1.37 (0.94-1.98) | .103 | **1** |
| ≥ 5000 to < 10000 | 0.82 | 2.26 (1.22-4.21) | .001 | **2** |
| ≥ 10000 | 0.83 | 2.30 (0.75-6.99) | .143 | **2** |
| OI (Ref = < 15) |  |  |  |  |
| ≥ 15 to < 25 | 0.17 | 1.18 (0.89-1.57) | .239 | **1** |
| ≥ 25 to < 35 | 0.55 | 1.73 (1.14-2.63) | .010 | **2** |
| ≥ 35 | 0.81 | 2.24 (1.21-4.17) | .011 | **2** |
| pH (Ref= > 7.30 to ≤ 7.40) |  |  |  |  |
| ≤ 7.10 | 0.99 | 2.70 (1.63-4.45) | <.001 | **3** |
| > 7.10 to ≤ 7.20 | 0.59 | 1.81 (1.22-2.69) | .003 | **2** |
| > 7.20 to ≤ 7.30 | 0.42 | 1.53 (1.12-2.08) | .007 | **2** |
| > 7.40 | 0.13 | 1.14 (0.80-1.62) | .48 | **1** |
| MAP/dose of needed vasopressor  (Ref=MAP ≥ 70) |  |  |  |  |
| MAP < 70, no vasopressor | 0.07 | 1.07 (0.75-1.52) | .70 | **1** |
| DOA ≤ 15 r/ EPI or NAD ≤ 0.1 r | 0.30 | 1.35 (0.96-1.92) | .09 | **1** |
| DOA >15 r/ EPI or NAD ≤ 0.2 r | 0.65 | 1.91 (1.20-3.02) | .006 | **2** |
| EPI or NAD > 0.2 r | 1.11 | 3.02 (1.95-4.68) | <.001 | **3** |

Abbreviations: y, years; CKD, chronic kidney disease; BUN, blood urea nitrogen; OI, oxygen index; Ref, reference level; Coef: coefficient; OR, odds ratio; CI, confidence interval; MAP, mean arterial pressure; r, mg/kg/min; DOA, dopamine; EPI, epinephrine; NAD, norepinephrine.

**eTable 4. Coefficients of all variables in the the Least Absolute Shrinkage Selection Operator regression model**

| **Variables** | **Coefficients** |
| --- | --- |
| Intercept | -0.280 |
| Age | 0.348 |
| Gender, male (vs. female) | 0.065 |
| BMI (vs <25) |  |
| ≥25 to <30 | -0.139 |
| ≥30 to <40 | -0.167 |
| ≥40 | -0.165 |
| Hypertension | -0.103 |
| Diabetes | 0.090 |
| Heart Disease | 0.165 |
| Lung Disease | 0.174 |
| Cancer | 0.018 |
| Dementia | -0.065 |
| CKD | 0.198 |
| Chronic Liver Diseases | -0.055 |
| Heart Rate, beats/min (vs. <70) |  |
| ≥70 to <90 | -0.165 |
| ≥90 to <110 | 0.100 |
| ≥110 to <130 | 0.000 |
| ≥130 | 0.029 |
| MAP/ dose of needed vasopressor (vs. MAP ≥70) |  |
| MAP <70, no vasopressor | -0.010 |
| DOA ≤15r/ EPI or NAD ≤0.1 r | 0.116 |
| DOA >15 r /EPI or NAD ≤0.2 r | 0.149 |
| EPI or NAD >0.2 r | 0.345 |
| Urine Output, ml/kg/h (vs. 0) |  |
| >0 to ≤0.3 | 0.054 |
| >0.3 to ≤0.5 | 0.085 |
| > 0.5 to ≤1.0 | 0.136 |
| >1.0 | -0.065 |
| Albumin, g/dL (vs. <2.8) |  |
| ≥2.8 to <3.5 | -0.060 |
| ≥3.5 | -0.120 |
| ALP, U/L | -.033 |
| Total Bil, mg/dL | 0.150 |
| Total protein, g/dL | 0.092 |
| BUN, mg/dL | 0.421 |
| Creatinine, mg/dL (vs. <1.2) |  |
| ≥1.2 to <2 | 0.199 |
| ≥2 to <3.5 | 0.019 |
| ≥3.5 to <5 | 0.051 |
| ≥5 | -0.046 |
| CRP, mg/L | 0.022 |
| D-dimer (vs. <5 times the upper limit of normal) |  |
| ≥5 times to <10 times | -0.070 |
| ≥10 times to <25 times | -0.014 |
| ≥25 times to <50 times | -0.089 |
| ≥50 times | 0.149 |
| Ferritin, x10^3^ ng/ml | 0.198 |
| Hematocrit (vs. <30) |  |
| ≥30 to <46 | 0.028 |
| ≥46 | 0.000 |
| NLR (vs. <10) |  |
| ≥10 to <20 | 0.063 |
| ≥20 to <30 | 0.141 |
| ≥30 to <40 | 0.106 |
| ≥40 | 0.031 |
| Plat Count, 10^5/^/uL (vs. <1) |  |
| ≥1 to <1.5 | 0.185 |
| ≥1.5 | 0.188 |
| Potassium, mmol/L (vs. <3.5) |  |
| ≥3.5 to <6 | -0.061 |
| ≥6 | 0.044 |
| Procalcitonin, ng/mL (vs. <0.5) |  |
| ≥0.5 to <2 | 0.046 |
| ≥2 to <10 | 0.005 |
| ≥10 | -0.072 |
| RCDW, % | 0.045 |
| Sodium, mmol/L (vs. <130) |  |
| ≥130 to <150 | 0.187 |
| ≥150 | 0.155 |
| WBC Count, K/uL (vs. <4) |  |
| ≥4 to <12 | -0.312 |
| ≥12 to <20 | -0.440 |
| ≥20 to <30 | -0.427 |
| ≥30 | -0.288 |
| Lactate, mmol/L | 0.164 |
| AaDO2 | 0.094 |
| Oxygen Index | 0.234 |
| HC03, mmol/L (vs. <15) |  |
| ≥15 to <18 | -0.053 |
| ≥18 to <22 | -0.203 |
| ≥22 to <32 | -0.192 |
| ≥32 | -0.199 |
| pH (vs. <7.10) |  |
| ≥7.10 to <7.20 | -0.035 |
| ≥7.20 to <7.30 | -0.182 |
| ≥7.30 to <7.40 | -0.379 |
| ≥ 7.40 | -0.229 |

Abbreviations: BMI, body mass index; CKD, chronic kidney disease; MAP, mean arterial pressure; r, mg/kg/min; DOA, dopamine; EPI, epinephrine; NAD, norepinephrine; ALP, alkaline phosphatase; Bil, bilirubin; BUN, blood urea nitrogen; CRP, C-reactive protein; NLR, neutrophil to lymphocyte ratio; Plat, platelet; RCDW, red blood cell distribution width; WBC, white blood cell.

**eFigures**

**eFigure Legends**

**eFigure 1. Frequency of Selection for Each Variable by Backward Elimination**

To create a predictive score, logistic regression models starting with all candidate predictors were fitted using each imputed set (33 sets). Backwards selection with selection entry *P* ≤.01 was applied. The variables appearing in at least half of the models (>17 times) were selected for our predictive score.

Abbreviations: WBC, white blood cell; BUN, blood urea nitrogen; MAP, mean arterial pressure; PI, oxygen index; BMI, CKD, chronic kidney disease; Bil, bilirubin; BMI, body mass index; Plat, platelet; RCDW, red blood cell distribution width; NLR, neutrophil to lymphocyte ratio; CRP, C-reactive protein; ALP, alkaline phosphatase.

**eFigure 2. Distribution of Predicted Probability of the ICOP Score**

The distribution of the ICOP score in both the deviation (A) and validation (B) cohorts.

**eFigure 3. Receiver Operating Characteristic Curves for 28-Day Mortality After Intubation**

The Receiver Operating Characteristic (ROC) Curves of our predictive scores (ICOP score and sICOP) for 28-day mortality after intubation in derivation (A) and validation (B) cohorts. The area under the curve (AUC) of the ICOP score was compared with SOFA score (C and D) and CURB-65 score (E and F) by DeLong’s test.

**eFigure 4. Area under the Receiver Operating Characteristic Curves for 14-Day Mortality After Intubation with the Least Absolute Shrinkage Selection Operator regression model**

Logistic regressions using the Least Absolute Shrinkage Selection Operator (LASSO regression) was also performed on the derivation set. The best-tuned model based on the area under the receiver operating curve after three repetitions of 10-fold cross validation yielded c=0.738.

**eFigure 1.**

**eFigure 2.**

**eFigure 3. Receiver Operating Characteristic Curves for 28-Day Mortality After Intubation**


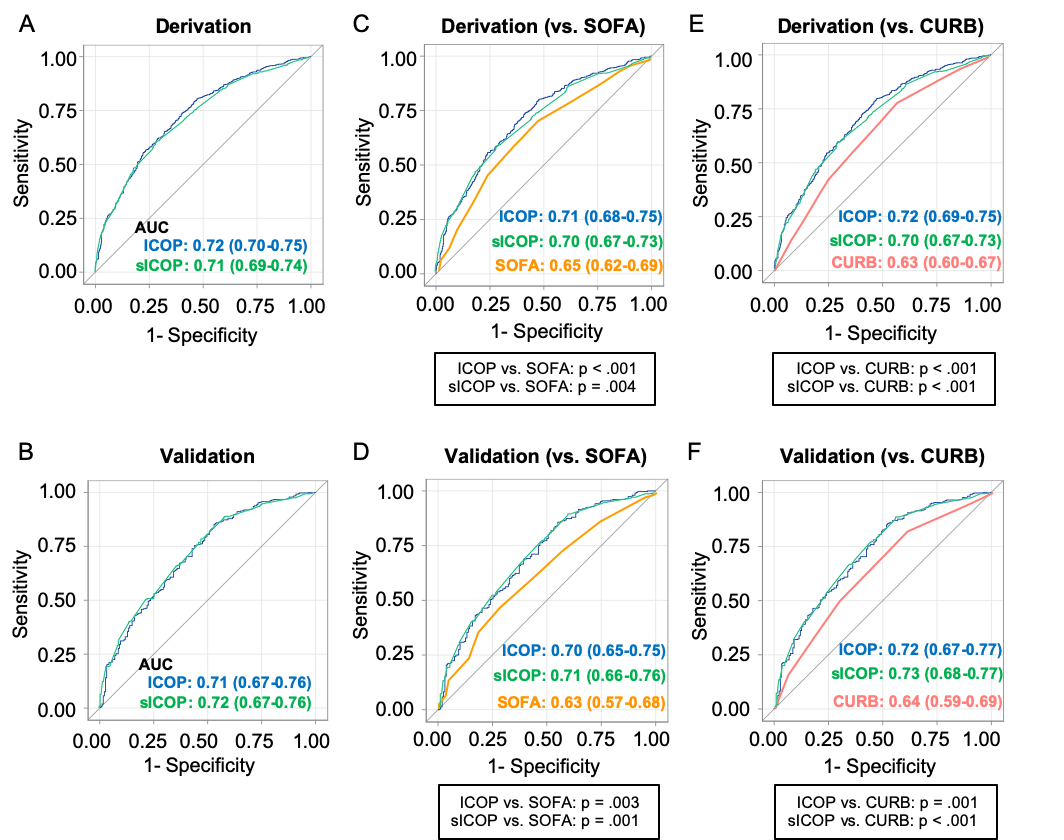


**eFigure 4. Repeated Cross-Validation Curve of each Receiver Operating Characteristic Curve (AUC) from each Least Absolute Shrinkage Selection Operator regression model Predicting 14-Day Mortality After Intubation along the Regularization Parameter Sequence**


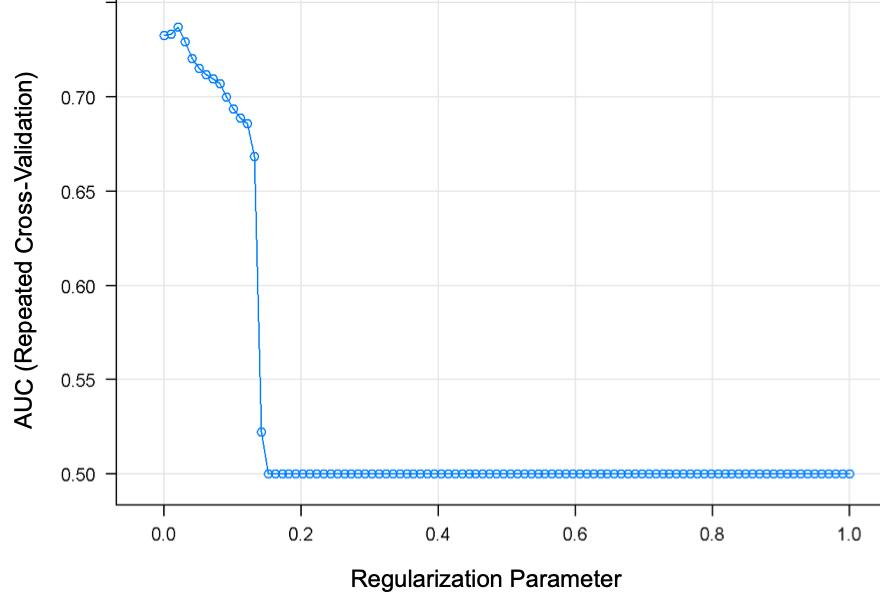

Supplement: Supplementary file 1 — Supplementary Information. [file 41598_2021_591_MOESM1_ESM.docx]
